# The role of intergenerational support in shaping oral healthcare-seeking behavior among older adults in China

**DOI:** 10.3389/fpubh.2023.1234539

**Published:** 2023-09-04

**Authors:** Cai Wen, Qing Zhang

**Affiliations:** ^1^Department of Oral Implantology, The Affiliated Stomatological Hospital of Southwest Medical University, Luzhou, Sichuan, China; ^2^Department of VIP Dental Service, The Affiliated Stomatological Hospital of Southwest Medical University, Luzhou, Sichuan, China; ^3^Luzhou Key Laboratory of Oral and Maxillofacial Reconstruction and Regeneration, The Affiliated Stomatological Hospital of Southwest Medical University, Luzhou, Sichuan, China; ^4^Institute of Stomatology, Southwest Medical University, Luzhou, Sichuan, China; ^5^Department of Nosocomial Infection Control, The Affiliated Hospital of Southwest Medical University, Luzhou, Sichuan, China

**Keywords:** oral health, older adults, CHARLS, intergenerational support, healthcare-seeking behavior

## Abstract

**Background:**

This study aimed to explore how intergenerational support affects the oral healthcare or treatment-seeking behaviors of older Chinese adults and provide evidence for improving the oral health of the older adults in an aging society.

**Methods:**

Data from a cross-sectional survey, the 2015 China Health and Retirement Longitudinal Study, were used to explore the relationship between oral healthcare-seeking behavior in older adults and various independent variables, such as marital status, number of children, offspring’s education duration, parent–offspring interaction frequency, and financial/material support provided by children. The chi-square test and binary logistic regression were used.

**Results:**

According to the results of data analysis, age, sex, marital status, cohabitation status, number of children, children’s education level, and financial support from children affected older adults’ oral healthcare-seeking behavior. Interviewees living with a partner and those who had 1–2 or 3–4 children showed different likelihoods of seeking oral healthcare. Moreover, interviewees whose children had higher education duration and those who received more financial/material support from their children were more likely to seek dental treatment.

**Conclusion:**

Regarding the study’s outcome, financial and emotional support, as well as practical assistance from family members can significantly promote oral health-seeking behavior among older adult people. Intergenerational support can serve as a crucial mechanism for promoting oral health behaviors among the older adults or act as a valuable complement to social medical assistance, warranting increased attention.

## 1. Introduction

As the world’s largest aging population, China had 264.02 million people aged over 60 years in November 2020, showing an increase of 5.44% over the course of one decade (from 13.26% in 2010 to 18.70% in 2020) according to the data from the Seventh National Population Census of China (1, 2). Since the 1980s, China has implemented a relatively strict family planning policy that has reduced the number of newborns and led to a significant decline in fertility rates (3). Simultaneously, more young people leave their hometowns to seek employment opportunities, and the number of young people who can stay in the area and communicate regularly with and care for older adults has decreased. This has increased the burden of maintaining health and welfare, including oral hygiene, among the older adult population.

According to the 8,020 Oral Health Movement, older adults aged 80 years or above should maintain at least 20 functional teeth (4, 5). However, the fact remains that oral health awareness among the older adult population is relatively weak for various reasons, and economic factors were considered to contribute to this (6, 7). Since dental treatments are typically not covered by insurance in China, individuals must bear the cost themselves. Insufficient retirement funds may deter some older individuals from seeking oral healthcare services due to the inability to cover associated expenses. Furthermore, inconvenient access to dental services for the older population may be another reason for their weaker oral healthcare-seeking behavior. Due to the rapid development and expansion of Chinese cities, many older adults living in rural areas face challenges accessing dental care in cities without assistance for transportation and navigation (8, 9). Intergenerational support from their younger offspring may provide a partial solution (10, 11).

Intergenerational support refers to the various forms of assistance adult children provide for their older adult parents, including financial aid, daily caregiving, and emotional comfort (12, 13). Owing to the imperfect formal social support system and limited self-financial resources available to older adults, intergenerational support from their offspring may become an important means to sustain themselves after retirement. Regarding oral health, older adults require the assistance and support of their offspring concerning financial capacity, treatment accessibility, and emotional well-being. Monetary aid from their adult children bolsters the fiscal capability of older adults concerning the medical services they can afford (14, 15). Caregiving by children enhances the ease of obtaining medical care for older adults, whereas emotional backing from offspring reflects the potential availability of medical resources for older adults (16).

**Figure 1:**
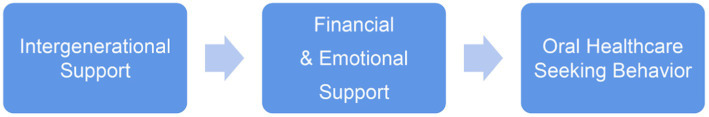
The logical diagram of the relationship between intergenerational support from adult children and Oral Healthcare seeking behavior of older adults.

In China, family care for older adults will remain the primary mode of care for a considerable period (17). Therefore, mutual healthcare support between generations cannot be ignored in terms of its effects on the oral health of older adults. Intergenerational support from adult children to parents may be crucial in promoting oral healthcare-seeking (OHS) behaviors in older adults (10, 18). Although the impact of intergenerational help in terms of economics, life, and emotions on the OHS of older adults may indirectly determine the degree of adequate medical service utilization, they have been relatively less studied (8, 18, 19) (Figure 1).

The China Health and Retirement Longitudinal Study (CHARLS) is a longitudinal survey project conducted in collaboration with the National Institute of Development and the China Social Science Survey Center of Peking University. The project aims to promote research on the health, lifestyle, and aging abilities of Chinese older adults and their families. Since 2011, the project has conducted biennial surveys on Chinese residents aged 45 and above, covering physical health, cognitive ability, psychological health, oral health, older adult healthcare arrangements, social networks, and more. By 2021, four rounds of surveys were completed nationwide in 28 provinces and over 450 counties and cities. Currently, it is one of the most critical data sources on the health and care of older adults in China (20, 21). In this study, we used data from the project to explore the impact of intergenerational support on the OHS behavior of older adults to provide theoretical support and empirical evidence to promote rational oral healthcare for older adults.

The older adult population has a high prevalence of oral diseases, uneven access to medical care, and expensive healthcare (6, 22, 23). With increasing electronic and mobile hospital registration, older adults face new challenges, particularly those who are unfamiliar with emerging technologies, such as smart mobile devices and electronic medical services (24, 25). Therefore, exploring how intergenerational support from adult children can enhance the oral healthcare service utilization of older adults is a topic of great importance. To the best of our knowledge, there are limited studies on this topic. This study aimed to investigate the role of such support in promoting OHS behavior among older adults by addressing emerging challenges and providing effective solutions for improving oral and medical care services for older adults, thereby enhancing their quality of life and health.

## 2. Materials and methods

### 2.1. Study design and impact analysis

**Figure 2:**
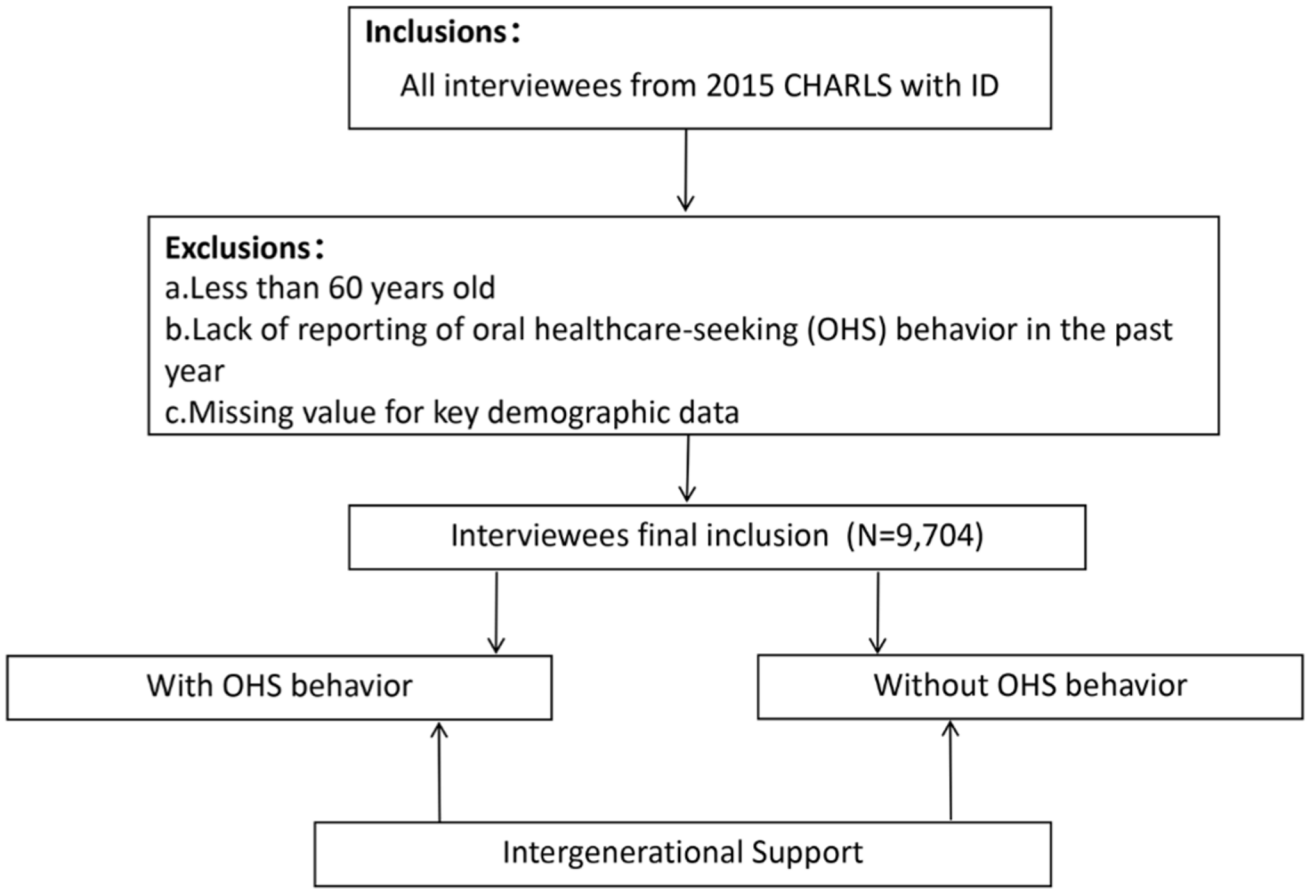
Flow chart of the inclusion screening process of Intergenerational Support on OHS behavior.

This study used cross-sectional data from an ongoing longitudinal study. Based on the question settings of surveys in different years, we selected the most recent survey out of the results relevant to this study—that is, CHARLS 2015. The study protocol was approved by the ethical review board of Peking University, and written informed consent was obtained from all participants. The exclusion criteria of interviewees were as follows: (1) interviewees who failed to report their OHS behavior data, (2) interviewees under the age of 60, and (3) interviewees with missing key demographic data. The flow chart of the study is shown in Figure 2.

### 2.2. Measurements

#### 2.2.1. Demographic and socioeconomic status

The assessment encompassed demographic variables, such as age and sex. Sex was dichotomized into men and women, whereas age was categorized into three groups: “60–69 years,” “70–79 years,” and “80 years or older.” Interviewees’ marital status was categorized into seven groups: married and living together, married but not living together, separated, divorced, widowed, never married, and cohabiting but unmarried. As some groups under-enrolled, the living status were categorized as either with a partner (married or unmarried cohabitation) or without a partner (married but not cohabiting, never married, widowed, divorced, or separated) to simplify the living conditions of cohabitation.

#### 2.2.2. Number of children

The number of living adult children each interviewee had was divided into four groups: 0, 1–2, 3–4, and 5 or more children.

#### 2.2.3. Children’s education duration

The average education level of the interviewee’s adult children was measured based on their education duration. To determine the mean education duration of one’s offspring, we added all of the interviewee’s children’s education durations together and divided by the number of children. The mean education duration for the older adults’ children was divided into six groups: (> 0, <= 3), (> 3, <= 6), (> 6, <= 9), (> 9, <= 12), (> 12, <= 16), and (> 16).

#### 2.2.4. Frequency of parent–child meetings

The frequency with which interviewees met with their children over the past year was collected. We categorized the interviewees’ parent–child meetings over the last year into five groups based on frequency: 0, 1–6, 7–36, 37–200, and 201+ meetings all together. The average number of meetings was calculated by dividing the total number of meetings by the total number of one’s children and grouped into five categories: 0, (> 0, <= 6), (> 6, <= 36), (> 36, <= 100), and (> 100).

#### 2.2.5. Intergenerational contacts

Intergenerational contact (e.g., phone calls, emails, instant messages) between the interviewees and their adult children in the past year was collected. We categorized the number of contacts into five groups: 0, 1–6, 7–36, 37–200, and 201+ times. Similar calculating method in the former factor, the average number of contacts was categorized into five groups: 0, (> 0, <= 6), (> 6, <= 36), (> 36, <= 100), and (> 100).

#### 2.2.6. Children’s financial support

The total amount of financial and material support the adult children provided to interviewees in the past year was collected. The support included cash support and in-kind support, which was converted into cash for calculation. This factor was grouped into five categories: 0, 1–2,000, 2,001–5,000, 5,001–20,000, and over 20,000 yuan (RMB).

#### 2.2.7. Analysis of the interviewee’s dental OHS behavior status

The OHS behavior of interviewees was assessed based on whether they had received dental treatment within the past year from the data in the 2015 CHARLS survey. The OHS behavior outcome is dichotomous, with options of either “yes” or “no.”

### 2.3. Statistical analysis

The analysis was performed using the Statistical Package for the Social Sciences 25.0 (SPSS Inc., United States). The relationship between intergenerational support and OHS behavior among older adults was analyzed. Different variables were evaluated using the chi-square test, including age, sex, marital and cohabitation status, number of children, children’s education duration, frequency of parent–child meetings, frequency of parent–child contact, and financial and material support received from children. Two-tailed *p*-values less than 0.05 were deemed statistically significant.

Logistic regression analysis was used to examine the association between intergenerational support and OHS behavior, with odds ratios accompanied by 95% confidence intervals (CI).

## 3. Results

### 3.1. Study participants

Based on the exclusion criteria, 9,704 interviewees (4,799 men and 4,905 women) were selected from the 2015 CHARLS for inclusion in the study. The proportion of women with OHS behavior (18.84%) in the last year was significantly higher than that of men (17.19%), and a chi-square test indicated that the difference was statistically significant (*p* = 0.035; Table 1).

**Table 1:** The proportion and variables related to older adult’s oral healthcare seeking behavior.

Variables	Dental visits in the past year		Total	Proportion %	*p*
	Yes	No			
*Gender*					0.035^*^
Men	825	3,974	4,799	17.19	
Women	924	3,981	4,905	18.84	
*Age*					0.000^*^
60-69	1,277	5,197	6,474	19.73	
70–89	404	2,121	2,525	16.00	
80–	68	637	705	9.65	
*Martial status*					0.012^*^
Married with spouse present	1,372	5,971	7,343	18.68	
Married but not living with spouse temporarily	50	259	309	16.18	
Separated	7	18	25	28.00	
Divorced	12	43	55	21.82	
Widowed	297	1,576	1873	15.86	
Never married	8	80	88	9.09	
Cohabitated	3	8	11	27.27	
*Cohabitation*					0.002^*^
Live with partner	1,375	5,979	7,354	18.70	
Live without partner	374	1976	2,350	15.91	
*Number of children*					0.000^*^
0	13	116	129	10.08	
1–2	434	1,637	2071	20.96	
3–4	501	2,213	2,714	18.46	
5–	157	958	1,115	14.08	
*Mean Years of Education per child*					0.000^*^
> = 0, <=3	27	250	277	9.75	
>3, <=6	200	1,268	1,468	13.62	
>6, <=9	399	1749	2,148	18.58	
>9, <=12	222	870	1,092	20.33	
>12, <=16	228	632	860	26.51	
>16	16	39	55	29.09	
*Total number of meetings*					0.085
0	86	384	470	18.30	
1–6	155	818	973	15.93	
7–36	168	827	995	16.88	
37–200	235	942	1,177	19.97	
201–	461	1953	2,414	19.10	
*Mean number of meetings per child*					0.056
0	75	275	350	21.43	
>0, <=6	206	1,063	1,269	16.23	
>6, <=36	222	998	1,220	18.20	
>36, <=100	220	868	1,088	20.22	
>100	339	1,407	1746	19.42	
*Total number of contacts*					0.137
0	341	1,592	1933	17.64	
1–6	31	195	226	13.72	
7–36	163	772	935	17.43	
37–200	406	1712	2,118	19.17	
201–	164	653	817	20.07	
*Mean number of contacts per child*					0.013^*^
0	330	1,477	1807	18.26	
>0, <=6	112	594	706	15.86	
>6, <=36	348	1,559	1907	18.25	
>36, <=100	285	1,062	1,347	21.16	
>200	17	116	133	12.78	
*Total financial support from children (currency & in-kind)*					0.001^*^
0	169	817	986	17.14	
1–2000	291	1,534	1825	15.95	
2001–5,000	267	1,140	1,407	18.98	
5,001–20,000	302	1,177	1,479	20.42	
20,001–	74	242	316	23.42	

^*^Indicate there was significant difference.

Among the interviewees, 6,474 were aged 60–69, 2,525 were aged 70–79, and 705 were over 80 years. The proportion of people with OHS behavior in older participants (over the age of 80) was significantly lower than that in younger participants, with a significant difference (*p* = 0.000).

Among those who reported their marital and cohabitation status, there were 7,343 married interviewees with spouse present, 309 married but temporarily not living with spouse, 25 separated, 55 divorced, 1,876 widowed, 88 never married, and 11 unmarried interviewees who cohabited with their partner. The proportion of interviewees with OHS behaviors varied significantly among the different groups according to a chi-square test (*p* = 0.012).

The proportion of OHS behavior between interviewees with and without a partner was evaluated. Of those with a living partner, 18.70% (7,354) reported OHS behavior last year compared with those without a cohabiting partner (2,350, 15.91%), and the difference was significant.

Regarding number of children of the surveyed older adults, 129 had none, 2,071 had 1–2, 2,714 had 3–4, and 1,115 had five or more. Significant differences were detected in OHS behavior proportions among these groups (*p* = 0.000). Those with no children had the lowest proportion of OHS behavior (10.08%), followed by those with five or more children (14.08%), whereas those with 1–2 children had the highest proportion (20.96%).

Regarding these interviewees’ children’s mean education duration, 277 had 0–3 years, 1,468 had 3–6 years, 2,148 had 6–9 years, 1,092 had 9–12 years, 860 had 12–16 years, and 55 had more than 16 years of education. Notable differences were detected in the proportions of OHS behavior among groups. A positive correlation was observed between adult children’s mean education duration and OHS rates.

Considering the frequency of meeting their children in the last year, 470 had no meetings (18.30%), 973 had 1–6 meetings (15.93%), 995 had 7–36 meetings (16.88%), 1,177 had 37–200 meetings (19.97%), and 2,114 had 201 or more meetings (19.10%). This study found no significant differences in the proportions of OHS behaviors among these groups. Similarly, the mean number of meetings with their parent per adult child also did not indicate a significant difference.

Considering contact between the adult children and their parents, there was no significant difference in the proportion of interviewees with OHS behaviors among different total contacts (*p* = 0.137). However, there was a significant difference among groups based on the mean contacts with their parent per child (*p* = 0.013), which were categorized as follows: (0) 18.26%, (> 0, <= 6) 15.86%, (> 6, <= 36) 18.25%, (> 36, < = 100) 21.16%, and (over 100) 12.78%.

There were significant differences in the proportion of older adult parents’ OHS behavior under different financial and material support (*p* = 0.001). The proportion of the older adults with OHS behavior was the lowest (15.95%) when their children gave 1–2000 yuan. After that, with the increase of the cost, the proportion of the older adults with OHS behavior was positively correlated and gradually increased, reaching the highest (23.42%) when they were above 20,001 yuan.

### 3.2. Logistic regression relationship between intergenerational support and OHS behavior

As shown in Table 2, the interviewees’ sex, marital status, cohabitation status, number of children, mean education of children, parent–children contact, and financial assistance provided by adult children were included in the in the binary logistic regression model for analysis.

**Table 2:** Results of binary logistic regression analysis of the association of Intergenerational support and oral healthcare seeking behavior.

	B	S.E.	Wald	df	P	OR	95%CI	
Variables								
*Gender*	−1.572	0.038	1688.559	1	0	0.208		
Men								
Women	0.112	0.053	4.45	1	0.035	1.118	1.008	1.240
*Age*	−1.404	0.031	2019.472	1	0	0.246		
60–69			51.426	2	0			
70–79	−0.255	0.063	16.534	1	0	0.775	0.686	0.876
80–	−0.834	0.131	40.29	1	0	0.434	0.336	0.562
*Martial status*	1.471	0.03	2412.922	1	0	4.352		
Married with spouse present			16.104	6	0.013			
Married but not living with spouse temporarily	0.174	0.157	1.225	1	0.268	1.19	0.874	1.620
Seperated	−0.526	0.446	1.389	1	0.239	0.591	0.246	1.417
Divorced	−0.194	0.328	0.351	1	0.553	0.823	0.433	1.566
Widowed	0.198	0.07	8.026	1	0.005	1.219	1.063	1.399
Never married	0.832	0.372	5.001	1	0.025	2.298	1.108	4.764
Cohabitated	−0.49	0.678	0.522	1	0.47	0.613	0.162	2.313
*Cohabitation*	−1.47	0.03	2415.037	1	0	0.230		
Live with partner								
Live without partner	−0.195	0.064	9.311	1	0.002	0.823	0.726	0.933
*Number of children*	−2.189	0.292	55.996	1	0	0.112		
0			28.411	3	0			
1–2	0.861	0.297	8.382	1	0.004	2.366	1.321	4.238
3–4	0.703	0.297	5.619	1	0.018	2.020	1.129	3.613
5–	0.38	0.305	1.554	1	0.213	1.462	0.805	2.658
*Mean Years of Education per child*	−2.226	0.203	120.706	1	0	0.108		
>0, <=3			77.956	5	0			
>3, <=6	0.379	0.216	3.063	1	0.080	1.46	0.956	2.232
>6, <=9	0.748	0.210	12.676	1	0	2.112	1.400	3.188
>9, <=12	0.86	0.216	15.833	1	0	2.363	1.547	3.609
>12, <=16	1.206	0.217	30.946	1	0	3.340	2.184	5.109
>16	1.335	0.359	13.789	1	0	3.799	1.878	7.684
*Total number of meetings*	−1.496	0.119	157.314	1	0	0.224		
0			8.166	4	0.086			
1–6	−0.167	0.148	1.275	1	0.259	0.846	0.633	1.131
7–36	−0.098	0.146	0.445	1	0.505	0.907	0.681	1.208
37–200	0.108	0.14	0.595	1	0.44	1.114	0.847	1.465
201–	0.053	0.13	0.163	1	0.686	1.054	0.817	1.36
*Mean number of meetings per child*	−1.299	0.13	99.479	1	0	0.273		
0			12.484	4	0.014			
>0, <=6	−0.376	0.148	6.418	1	0.011	0.687	0.514	0.918
>6, <=36	−0.204	0.15	1.848	1	0.174	0.816	0.608	1.094
>36, <=100	−0.073	0.151	0.237	1	0.626	0.929	0.692	1.248
>100	−0.124	0.144	0.744	1	0.388	0.883	0.667	1.171
*Total number of contacts*	−1.541	0.06	666.798	1	0	0.214		
0			6.944	4	0.139			
1–6	−0.298	0.202	2.171	1	0.141	0.742	0.499	1.103
7–36	−0.014	0.105	0.019	1	0.891	0.986	0.803	1.211
37–200	0.102	0.081	1.568	1	0.21	1.107	0.944	1.298
201–	0.159	0.106	2.264	1	0.132	1.173	0.953	1.443
*Mean number of contacts per child*	−1.496	0.061	604.881	1	0	0.224		
0			7.456	4	0.114			
>0, <=6	−0.173	0.12	2.085	1	0.149	0.841	0.666	1.064
>6, <=36	−0.004	0.085	0.002	1	0.963	0.996	0.843	1.176
>36, <=100	0.167	0.099	2.828	1	0.093	1.182	0.973	1.435
>100	0.069	0.129	0.283	1	0.595	1.071	0.832	1.379
*Total financial support from children (currency & in-kind)*	−1.576	0.085	347.697	1	0	0.207		
0			18.058	4	0.001			
1–2000	−0.088	0.106	0.684	1	0.408	0.916	0.745	1.127
2001–5,000	0.124	0.108	1.311	1	0.252	1.132	0.915	1.400
5,001–20,000	0.215	0.106	4.107	1	0.043	1.240	1.007	1.528
20,001–	0.391	0.157	6.164	1	0.013	1.478	1.086	2.013

OR, odds ratio; CI, confidence interval; S.E., standard error.

In terms of differences due to gender, women interviewees had a higher likelihood of OHS behavior (OR = 1.118, 95%CI = 1.008, 1.240) compared with men.

Among interviewees categorized by marital status, widowed and never married individuals exhibited a higher propensity for engaging in OHS behavior compared to interviewees who were married with their spouse present. However, it is important to note that the sample sizes of these respective groups varied significantly. In the simplified comparison between those with and those without a partner, individuals without a partner exhibited lower levels of OHS behavior (OR = 0.823, 95% CI = 0.726–0.933).

Interviewees with 1–2 children (OR = 2.366, 95% CI =1.321, 4.238) and 3–4 children (OR = 2.020, 95% CI = 1.129, 3.613) had higher likelihoods of seeking oral healthcare service than those without children. Interviewees whose children had a longer duration of education (with 6–9, 9–12, 12–16, or > 16 years of education) were more likely to exhibit OHS behavior than those with a lower education duration (0–3 years).

Interviewees who received financial and material support of 5,001–20,000 yuan (OR = 1.240, 95% CI = 1.007, 1.528) or more than 20,001 yuan (OR = 1.478, 95% CI = 1.086, 2.013) from their adult children were more likely to seek OHS compared with those who did not receive any support from their children.

## 4. Discussion

Older adults may encounter challenges in seeking oral healthcare service and treatment owing to their limited mobility, financial constraints, and lack of familiarity with electronic devices (26). This study aimed to investigate the potential of intergenerational support in facilitating dental care for older adults. Notably, factors such as individuals with a living partner, the frequency of adult children’s contact, and interaction with their parents, along with their intergenerational provision of financial and material aid, made significant difference on the OHS behavior proportion of these interviewees. This supports the idea that intergenerational support influences older adults’ decisions regarding their OHS behavior.

Older adults are vulnerable to health and economic issues, leading to potential health expenditures. It was found that the children’s intergenerational and emotional support will increase when catastrophic health expenditures occur in families with older adults (14). Furthermore, studies by Li et al. (17) and Yang et al. (27) also indicate that providing intergenerational support, encompassing both emotional and financial assistance, can have a positive impact on the health and well-being of older parents.

Intergenerational support can come from different family members. Liang et al. (28) compared intergenerational support from children with that from spouses and siblings, and results indicated that support from spouses and siblings did not have a significant impact but emphasized the importance of support from different generations of children for older adults’ well-being. Similarly, our study also focuses on family support from different generations.

### 4.1. Intergenerational support and modern technology

In addition to economic problems, with the continuous development of modern medical technology, many network medical services and mobile health solutions have entered medical institutions (29). This equipment and services bring a more intelligent and convenient experience to the medical treatment process (30–32). Additionally, mobile apps enable users to register and pay fees without having to physically visit medical institutions or wait in queues, thereby enhancing the convenience and efficiency of accessing healthcare services. However, owing to physical and cognitive decline, older adults may face difficulties in using high-tech and network medical service.

They may be unfamiliar with and unskilled in using computers, smartphones, and so on, have difficulty making online medical appointments, and be unfamiliar with hospital routes and navigation equipment.

Therefore, owing to today’s societal transition, the development of online medicine, and the lack of familiarity of the older adults with modern smart and mobile devices, intergenerational support by children may also play an important role in promoting the convenience of medical treatment and OHS behavior for older adults. This presents society with challenges: how to leverage intergenerational support from adult children to assist these older adults as well as how to enhance accessibility for those who lack such support through optimizing service processes.

### 4.2. Number of children and their education duration

The number of offspring affects the availability of caregivers and financial support for older adults during illness. Having more children may increase the likelihood of obtaining assistance when seeking medical treatment. This is consistent with the concept of raising children for help in old age in traditional Chinese culture (17).

Oral healthcare may be an important but neglected area in the care of the older adults. As individuals age, dental health becomes increasingly significant owing to its association with various health issues such as diabetes, heart disease, and dementia. Having a larger number of offspring can be particularly advantageous in ensuring adequate oral healthcare for older adults. This is because dental caregiving often requires both physical and emotional support, and having more caregivers available can help ensure that the necessary assistance is provided. For instance, family members may need to provide transportation to dental appointments, assist with oral hygiene maintenance, and help manage any dental-related anxieties that may arise. Involving more family members in oral healthcare and facilitating the mutual exchange and acquisition of more skills and expertise may also be required (33).

From the statistical results of this big data, it seems that a greater number of offspring may play a role in ensuring the oral health of older adults. With more potential caregivers and supporters available, they can receive the necessary physical and emotional support to maintain good dental health, thus improving long-term overall health outcomes.

However, the number of children reflects only the potential number of caregivers, and it requires a series of other factors for potential caregivers to become caregivers. These factors include children’s filial piety and socioeconomic status. In traditional agricultural societies, children are encouraged to meet the needs of the rural labor force. However, in modern society with improved living standards and education levels, people advocate for better quality rather than quantity of children, emphasizing the importance of nurturing them effectively (34).

As there may be a positive correlation between children’s levels of education and their socioeconomic status (35), regarding intergenerational support, children with higher education levels are more likely to have a high socioeconomic status and personal skills, which increases their likelihood of providing sufficient support to their parents (36). Well-educated children may increase their own awareness of oral diseases, which is helpful in urging parents to take appropriate treatment measures and guide them to conduct OHS behavior (34).

### 4.3. Intergenerational relationships and OHS

In addition to the quantity and education characteristics of offspring, intergenerational relationships are key factors in determining intergenerational support (37). To some extent, the frequency of intergenerational interactions reflects the state of these relationships. Therefore, when examining the impact of intergenerational support on older adult oral healthcare behaviors, we included the frequency of older adults meeting and communicating with their children as independent variables.

The decline of cognitive factors and self-care ability in the older adults may impact their medical treatment-seeking behaviors, necessitating the accompaniment and care of their offspring (38). Therefore, intergenerational support for healthcare is crucial. Increased contact between older adults and their children can encourage the latter to seek medical treatment for oral diseases and also accompany their parents during healthcare visits. Therefore, the degree of intimacy in intergenerational relationships may positively impact older adults’ OHS behavior.

Currently, with China’s urbanization, many youths from small cities flock to big cities such as provincial capitals, resulting in limited opportunities for parents and children to meet. The relationship between adult children and parents can be characterized by prolonged periods of no contact, even in cases when the parent is ill. In other instances, although children may maintain some level of communication with their parents, they cannot provide assistance due to living a great distance away. Though the results for the effect of parent–adult children meetings or contact on OHS behavior showed no significant difference in this study, there was still a trend: except for some older adults who did not have contact with their children at all, the proportion of the rest of older adults with OHS behavior increased with the number of contacts and meetings with their children.

### 4.4. Intergenerational financial support and OHS

Medical expenses for older adults constitute a substantial portion of overall expenditure (14, 39). Currently, most oral diseases in China are not covered by medical insurance. Given their limited income and insufficient pension, the willingness of older adults to seek oral treatment may be closely related to their children’s financial support.

From a legal perspective, adult children are not mandated to provide financial assistance to their parents. However, in many cultures, support and care for parents are considered part of filial piety. Many adult children recognize the dedication and sacrifice of their parents during their upbringing and wish to repay them. This type of mutual aid relationship is very important for building a harmonious family environment and promoting family cohesion. The provision of a living stipend or assistance to parents in distress can alleviate the financial burdens of daily life, encompassing medical expenses, housing costs, and other essential expenditures. Through such support, children can ensure that their parents’ fundamental needs are met and provide them with a secure and comfortable living environment.

Although older adults may sustain themselves without financial assistance from their children while in good health, their economic situation can worsen when they fall ill, and dental treatments for older adults, such as addressing dentition defects, can be expensive (40, 41). However, when children provide financial and in-kind support to their older-adult parents, it positively impacts their parents’ OHS behavior.

The result of this study confirmed that there was a positive correlation between financial and in-kind support from adult children to their parents and more frequent OHS behaviors by the parents. This not only addresses the financial constraints that older adults may face but also promotes better access to dental care, improves attitudes toward oral health, and encourages proactive oral health practices.

### 4.5. Limitations

First, the retrospective nature of the survey report from the CHARLS may introduce recall bias among interviewees, as they may struggle to accurately recollect past events or overestimate or underestimate their impact. Additionally, self-report surveys are susceptible to social desirability bias: although the interviewees were informed of the nature of the survey before their participation, they may have chosen to withhold information about their economic circumstances and specific financial support received from their offspring.

Second, a dichotomous response for the use of oral healthcare treatment as an indicator of OHS behaviors may have limitations. Due to limitations in the design of the CHARLS survey, there are missing results for the number and cost of oral treatments, with many confounding factors present. Therefore, this dichotomous result remains the best available evidence for understanding OHS behavior. OHS behavior may need to be combined with other indicators, such as oral health-related quality of life, to provide a more comprehensive overview of oral health status in older adults.

## 5. Conclusion

This study presents evidence of the relationship between intergenerational support and OHS behaviors among older adults in Chinese families. This study identified factors such as the number of children’s education duration, the frequency of parent–child contact, and economic assistance provided to parents positively connect with these older adults’ OHS behaviors.

With the increasing trend of population aging, older adults encounter numerous difficulties and challenges in accessing oral healthcare treatment. Recognizing the crucial role of intergenerational support from adult children and family members in maintaining the oral health of older adults is essential for establishing an age-friendly dental care system.

## Data availability statement

Publicly available datasets were analyzed in this study. These data are open and available at CHARLS project 2015 (http://charls.pku.edu.cn/).

## Ethics statement

This study used data from China Health and Retirement Longitudinal Study (CHARLS). The CHARLS project team received approval from the Ethical Review Committee of Peking University in January 2011. All participants signed the informed consent.

## Author contributions

CW: conceptualization, resources, writing-original draft preparation, writing-review and editing, supervision, project administration, and funding acquisition. QZ: validation, investigation, and formal analysis. All authors contributed to the article and approved the submitted version.

## Funding

This research was funded by grants from the Teaching Reform Project of Standardized training of residents from Affiliated Stomatological Hospital of Southwest Medical University (No. 2022GP04), the Innovation and Entrepreneurship Training Program of Southwest Medical University (Nos. 2022074 and 2022045).

## Conflict of interest

The authors declare that the research was conducted in the absence of any commercial or financial relationships that could be construed as a potential conflict of interest.

## Publisher’s note

All claims expressed in this article are solely those of the authors and do not necessarily represent those of their affiliated organizations, or those of the publisher, the editors and the reviewers. Any product that may be evaluated in this article, or claim that may be made by its manufacturer, is not guaranteed or endorsed by the publisher.
